# Caveolin-1 regulates the expression of tight junction proteins during hyperoxia-induced pulmonary epithelial barrier breakdown

**DOI:** 10.1186/s12931-016-0364-1

**Published:** 2016-05-12

**Authors:** Shuyan Xu, Xindong Xue, Kai You, Jianhua Fu

**Affiliations:** Department of Pediatrics, Shengjing Hospital of China Medical University, Shenyang, 110004 Liaoning China

**Keywords:** Caveolin-1, Tight junction protein, Pulmonary epithelial barrier, Hyperoxia, Bronchopulmonary dysplasia

## Abstract

**Background:**

Bronchopulmonary dysplasia (BPD) is a common complication in preterm infants that involves the downregulation of tight junction (TJ) proteins. However, the mechanism underlying downregulation of the expression of TJ proteins during at the early stages of hyperoxia-induced BPD remains to be understood. Here, we aimed to identify the role of caveolin-1 (Cav-1) in hyperoxia-induced pulmonary epithelial barrier breakdown.

**Methods:**

First, we established an in vitro pulmonary epithelial barrier models using primary type II alveolar epithelial cells (AEC-II) from newborn rats. AEC-II was assigned to the hyperoxic (85 % O_2_/5 % CO_2_) or normoxic (21 % O_2_/5 % CO_2_) groups. Second, AEC-II was transfected with Cav-1-siRNA to downregulate Cav-1 under normoxic exposure. Third, AEC-II was transfected with a cDNA encoding Cav-1 to upregulate Cav-1 expression under hyperoxic exposure. Then, expression levels of Cav-1 and TJ proteins were examined by immunofluorescence staining, reverse transcription-polymerase chain reaction, and Western blotting. The TJ structures visualized using a transmission electron microscope, and transepithelial resistance and apparent permeability coefficient of fluorescein isothiocyanate–dextran, which are indicators of barrier function, were measured.

**Results:**

Our data showed that exposure to hyperoxia disrupted the structure and function of the pulmonary epithelial barrier and decreased the ZO-1, occludin, claudin-4, and Cav-1 expression levels. Moreover, Cav-1 knockdown attenuated the expression of the other three genes and disrupted pulmonary epithelial barrier structure and function under normoxic exposure. However, Cav-1 upregulation markedly antagonized the hyperoxia-induced pulmonary epithelial barrier destruction and TJ protein loss.

**Conclusions:**

This is the first study to present evidence illustrating the novel role of Cav-1 downregulation-mediated TJ protein loss in pulmonary epithelial barrier destruction during BPD.

**Electronic supplementary material:**

The online version of this article (doi:10.1186/s12931-016-0364-1) contains supplementary material, which is available to authorized users.

## Background

Bronchopulmonary dysplasia (BPD) is one of the most severe complications in preterm infants, which is a lung injury that occurs due to high oxygen exposure and mechanical ventilation [1]. Apart from hyperoxia and mechanical ventilation, infection and preterm birth are also involved in causing BPD [2]. Although the occurrence of severe BPD has been reduced due to advances in clinical treatment, improvements in mechanical ventilation, and the clinical application of lung surfactant and glucocorticoids in preterm infants, the incidence remains to be high in very low birth weight infants [3]. Infants with BPD require long-term oxygen supplementation after hospitalization, as BPD is associated with potential multiple complications in the respiratory and nervous system [4, 5]. Therefore, there is an urgent need to understand the cause of BPD and use this information to develop prevention and treatment strategies.

The mechanism of BPD remains to be fully understood; however, an increasing body of evidence indicates that the main pathological changes include early-stage pulmonary edema [6–8] and late-stage defects in alveolar and microvasculature development [9]. Current research mainly focuses on these defects, but the mechanism of pulmonary edema in BPD has not been studied in detail. In our previous study, we used a hyperoxia-induced BPD model and showed that BPD is accompanied by destruction of TJ structures in the lung, increased permeability of the pulmonary epithelial barrier, and a decline in the concentrations of certain TJ proteins [7]. Thus, it is quite possible that the pulmonary edema at an early stage of BPD resulted from the destruction of structure and function of pulmonary epithelial barrier caused by the loss of TJ proteins.

TJ is a protein complex among neighboring cells, which includes integral proteins such as occludin, claudins, and tricellulin, and adaptor proteins like cingulin and ZOs [10]. Previous research has shown that ZO-1 may serve as a link between the TJ proteins such as occluding and the actin cytoskeleton [11]. ZO-1, occludin, and claudin-4 are important components of TJ in pulmonary epithelial barrier [12]. Occludin is a type of transmembrane protein and is required for maintaining the integrity of lung epithelial barrier [13, 14]. ZO-1 is a scaffold protein that promotes the assembly of protein–protein complex and influences the structure and function of lung epithelial barrier [15, 16]. Claudin-4 is also a type of transmembrane protein that improves the barrier function of pulmonary epithelial barrier by promoting pulmonary fluid–clearance function [17, 18]. In our previous study, in the hyperoxia-induced BPD model, TJ protein ZO-1 and occludin were both found to decrease, and it was therefore predicted that this may be the mechanism underlying of hyperoxia-induced destruction of lung epithelial barrier [7]. Therefore, we aimed to analyze the expression of ZO-1, occludin, and claudin-4 in relation to caveolin-1 in this study.

Tight junction (TJ) protein expression and assembly are regulated by several factors such as tumor necrosis factor-α (TNF-α), interferon-γ (IFN-γ) [19], matrix metalloproteinases (MMPs) [20], microRNAs [21], small GTPases [22], and phosphorylation-related regulation [23]. Caveolin-1 (Cav-1) is an important structural and regulatory component of caveolae, which is involved in multiple physiological processes including vesicle transportation and cellular signal transduction [24, 25]. Cav-1 has also been indicated to be involved in regulating the assembly of TJ proteins [26] and further influencing the function of blood–brain barrier [27] and intestinal epithelial barrier [28]. In the lung, Cav-1 is highly expressed in epithelial cells, fibroblast, vascular endothelial cells, and inflammatory cells [29]. Cav-1 downregulation in lung tissue has been shown to be highly correlated with BPD [30]. Gao et al. reported that pulmonary injection of Cav-1-siRNA could increase the permeability of the pulmonary epithelial barrier in rats [31], but the underlying mechanism remains to be elucidated. Therefore, we used an in vitro pulmonary epithelial barrier model to investigate the role of Cav-1 in hyperoxia-induced destruction of the pulmonary epithelial barrier, and to elucidate the molecular mechanisms underlying pulmonary epithelial barrier destruction in BPD. The results of this study provide a theoretical basis for preventing and treating pulmonary edema at an early stage of BPD.

## Methods

### Isolation of type II alveolar epithelial cells (AEC-II)

Pregnant Wistar rats weighing 200–220 g were purchased from the Experimental Animal Center of China Medical University. Each pregnant Wistar rat was independently feeding, and gave birth to litters via natural birth at 22 days of pregnancy. AEC-II was isolated as described previously [6]. Within 12 h of birth, newborn Wistar rats were anesthetized by intraperitoneal injection of 10 % chloral hydrate (3 mL/kg). After cardiopulmonary lavage, the lung tissues were dissected and sectioned into 1-mm^3^ pieces in a sterile condition. The samples were digested in 0.25 % trypsin EDTA (Gibco Life Technologies, Grand Island, NY, USA) followed by filtration, and further digested with 0.1 % collagenase I (Gibco Life Technologies). The cells were harvested by centrifugation at 800 rpm, resuspended and incubated in a 5 % CO_2_ incubator at 37 °C. To remove fibroblasts, the medium was poured every 1 h for three times. To remove macrophages, nonadherent cells were transferred to rat immunoglobulin G (IgG)-coated plates and incubated for 1 h. The viability (>95 %) and purity (>90 %) of AEC-II were tested by trypan blue staining and immunostaining of AEC-II-specific marker surfactant protein C (SP-C), respectively.

All procedures and animal experiments were approved by the Animal Care and Use Committee of China Medical University.

### Establishment of an in vitro pulmonary epithelial barrier model

Based on previous protocol for establishing an in vitro pulmonary epithelial barrier model [32, 33], AEC-II was seeded onto Transwell Clear inserts (diameter, 6.5 mm; pore size, 0.4 μm; Corning Costar, Cambridge, MA, USA) at a density of 2–3 × 10^6^ cells/mL and cultured in Dulbecco’s modified Eagle’s medium (DMEM)/F12 (Hyclone, Logan, UT, USA) with 10 % fetal bovine serum (FBS; Clark, Seabrook, MD, USA). The medium was changed after 24 h. The pulmonary epithelial barrier could be successfully established after consecutive in vitro culture for 7 days and then randomly grouped into hyperoxic and normoxic groups. The hyperoxic group was incubated in an 85 % O_2_/5 % CO_2_ incubator (CB150, Binder, Tuttlingen, Germany), while the normoxic group was incubated in a 21 % O_2_/5 % CO_2_ incubator (3111, Thermo Fisher Scientific, Marietta, OH, USA). Cells were collected for RT-PCR, Western blot and immunofluorescence staining, and simultaneously, pulmonary epithelial barrier function was measured after 0, 24, 48, and 72 h under exposure to hyperoxic or normoxic conditions.

### Transfection of primary cultured AEC-II

Primary cultured AEC-II were seeded onto Transwell Clear inserts and cultured to 60–70 % confluence before transfection. For Cav-1-siRNA transfection, cells were transfected with 10 nm of a rat Cav-1-specific siRNA (5’-GGCAUAGCACAAGUAAUAGUCUGTA-3’; Cat.# SR500200; OriGene Technologies, Rockville, MD, USA) or 10 nm of a non-silencing scrambled control siRNA (Cat.#SR30004;OriGene Technologies) using a siTran 1.0 transfection reagent (OriGene Technologies), according to the manufacturer’s protocol. For Cav-1-cDNA transfection, cells were exposed to hyperoxia (85 % O_2_ and 5 % CO_2_) and transfected with Cav-1 cDNA plasmid (1 μg/mL; Cat.#RR200414; OriGene Technologies) or with an empty vector plasmid (1 μg/mL; Cat.#PS100001; OriGene Technologies) as control using Turbofectin 8.0 transfection reagent (OriGene Technologies), following the manufacturer’s instructions.

Cav-1 mRNA and protein expression levels were monitored using reverse transcription-polymerase chain reaction (RT-PCR) and Western blot analysis, respectively, to determine the best concentration of Cav-1-siRNA or Cav-1 cDNA plasmid for transfection and the best post-transfection time for the lowest and highest Cav-1 expression. Cells transfected with siRNA or cDNA plasmid were harvested at 48 and 72 h after transfection, respectively, before further experiments.

### RT-PCR

The total RNA was extracted from pulmonary epithelial cells using Trizol reagent (Invitrogen, Camarillo, CA, USA), and the purity was determined by spectrophotometer (OD_260_/OD_280_). The total RNA (1 μg) was reverse-transcribed to cDNA using SuperScript III First-Strand Synthesis System (Invitrogen). Primers were designed and synthesized by TaKaRa Inc. (Shiga, Japan) as follows: Cav-1, forward: 5’-CGGGAACAGGGCAACATCTAC-3’, reverse: 5’-CTTCTGGTTCCGCAATCACATC-3’; ZO-1, forward: 5’-CCATCTTTGGACCGATTGCTG-3’, reverse: 5’-TAATGCCCGAGCTCCGATG-3’; Occludin, forward: 5’-GTCTTGGGAGCCTTGACATCTTG-3’, reverse: 5’-GCATTGGTCGAACGTGCATC-3’; Claudin-4, forward: 5’-ACGAGACCGTCAAGGCCAAG-3’, reverse: 5’-GTCCAGGACACAGGCACCATAA-3’; β-actin, forward: 5’-GGAGATTACTGCCCTGGCTCCTA-3’, reverse: 5’-GACTCATCGTACTCCTGCTTGCTG-3’. The conditions of amplification were as follows: 30 cycles at 95 °C for 30 s, 55 °C for 40 s, and 75 °C for 1 min in a thermal cycler (ABI, Vernon, CA, USA). The PCR amplicons were densitometrically analyzed by 2.5 % agarose gel electrophoresis using ImageJ software (National Institutes of Health, Bethesda, MD, USA). The relative expression level of target genes was calculated after normalization to the levels of β*-actin*.

### Western blot analysis

Briefly, equal amounts of protein were extracted from pulmonary epithelial cells and separated by electrophoresis on 10 % sodium dodecyl sulfate (SDS) polyacrylamide gel and transferred to polyvinylidene difluoride (PVDF) membranes (Millipore, Billerica, MA). After blocking with 5 % bovine serum albumin (BSA), the PVDF membranes were incubated with the following primary antibodies at 4 °C overnight: rabbit anti-rat polyclonal antibody against Cav-1 (1:1000, Cat.#3238, Cell Signaling, Danvers, MA), rabbit anti-rat polyclonal antibody against ZO-1 (1:500, Cat.#40-2200, Invitrogen, San Francisco, CA, USA), rabbit anti-rat polyclonal antibody against occludin (1:500, Cat.#71-1500, Invitrogen), mouse anti-rat monoclonal antibody against claudin-4 (1:500, Cat.#32-9400, Invitrogen), and rabbit anti-rat polyclonal antibody against β-actin (1:1000, Cat. sc-130657, Santa Cruz Biotechnology, CA, USA). After washing, the membranes were incubated with horseradish peroxidase-conjugated secondary antibody (Cell Signaling, Beverly, MA, USA). The membranes were washed again and visualized with chemiluminescence substrate (ECL kit; Santa Cruz Biotechnology). The optical density of the protein bands was analyzed using ImageJ software and normalized to that of β-actin.

### Immunofluorescence staining

Pulmonary epithelial cells were fixed in 4 % paraformaldehyde for 30 min and washed thrice with phosphate-buffered saline (PBS). After blocking with 10 % goat serum, cell monolayers were incubated with the following primary antibodies: rabbit anti-rat polyclonal antibody againstZO-1 (1:100), rabbit anti-rat polyclonal antibody against occludin (1:100), and mouse anti-rat monoclonal antibody against claudin-4 (1:50). For double immunofluorescence staining, cells were incubated with a mixture of two primary antibodies as follows: rabbit anti-rat polyclonal antibody against ZO-1 (1:100) were mixed with mouse anti-rat monoclonal antibody against Cav-1 (1:100, Cat.ab17052, Abcam, New Territories, HK), rabbit anti-rat polyclonal antibody against occludin (1:100) were mixed with mouse anti-rat monoclonal antibody against Cav-1 (1:100), and mouse anti-rat monoclonal antibody against claudin-4 (1:50) were mixed with rabbit anti-rat polyclonal antibody againstCav-1 (1:100). The primary antibodies were incubated overnight at 4 °C. The cells were then washed and incubated with donkey anti-mouse IgG (H + L) secondary antibody, Alexa Fluor 594 conjugate (1:100, Invitrogen), and donkey anti-rabbit IgG (H + L) secondary antibody, and Alexa Fluor 488 conjugate (1:100, Invitrogen). The cells were washed again and images were obtained using a confocal laser scanning microscope (MTC-600, Bio-Rad, CA, USA) at 800× magnification.

### Transmission electron microscopy

The TJ structures in the in vitro pulmonary epithelial barrier were examined by transmission electron microscopy (TEM) as described in a previous study [34]. Pulmonary epithelial cells were fixed with 2 % paraformaldehyde and 2 % glutaraldehyde at 4 °C overnight. After washing with PBS and post-fixation in 1 % osmium tetroxide in phosphate buffer (pH 7.4) for 1 h, the cells were dehydrated in a graded ethanol series and embedded in the epoxy resin media. The resin blocks were then cut into ultrathin sections (60 nm) with a diamond knife, stained with uranyl acetate and lead citrate for contrast, and viewed by TEM (JEM-1200EX, Hitachi Electronic Company, Tokyo, Japan) at 20,000× or 80,000× magnification.

### Measurement of transepithelial electrical resistance

Transepithelial electrical resistance (TEER) was measured with a Millicell ERS volt-ohmmeter (Millicell ERS-2,Millipore, MA, USA). The average TEER was calculated by subtracting the average resistance of the cell-free culture inserts and corrected for the area covered by the cell monolayer. Resistance of a unit area = Resistance (Ω) × Effective membrane area (cm^2^) [35].

### Measurement of cell monolayer permeability

The permeability of cell monolayers was measured with the apparent permeability coefficient (P_app_) of fluorescein isothiocyanate–dextran (MW 4400, FD4). Briefly, FD4 (Sigma-Aldrich, St Louis, MA, USA) was directly added to the apical compartments of Transwell inserts (50 mg/mL in PBS), and its fluorescence in the basolateral compartments was measured using a spectrofluorometer (F-2000, Hitachi, Tokyo, Japan) every 30 min for 2 h at excitation and emission wavelengths of 495 and 515 nm. Hank’s buffered salt solution (HBSS, pH 7.4) was used as the solution in the basolateral compartments. P_app_ was calculated as follows:

$$ \mathrm{Papp} = \frac{dQ/dt}{A\times {C}_0} $$

Where *dQ*/*dt* is the FD4 transfer rate, *A* is the surface area of the Transwell membrane, and *C*_0_ is the initial concentration of FD4 in the apical compartment [36].

### Statistical analysis

Statistical analysis was performed using SPSS 17.0 software (IBM SPSS Inc., Chicago, USA). Data were presented as the means ± standard deviation (SD). An unpaired Student’s *t-*test was used to determine the significant differences between the different groups. One-way analysis of variance (ANOVA) and Bonferroni tests were used to determine the intragroup significant difference at different time points. A *P* value of <0.05 was considered to be statistically significant.

## Results

### Hyperoxia exposure impairs the structure and function of the in vitro pulmonary epithelial barrier

**Fig. 1:**
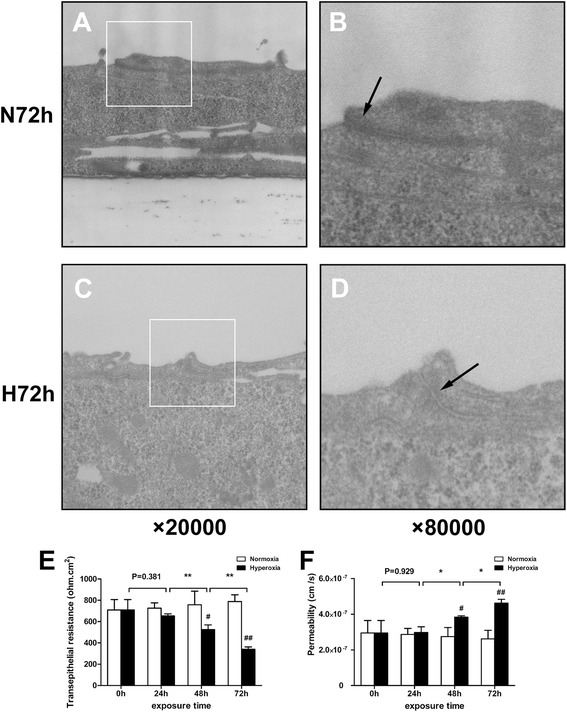
Effects of hyperoxia exposure on the structure and function of the pulmonary epithelial barrier in vitro*.* Alveolar cell monolayers were exposed to normoxia (N) (21 % O_2_/5 % CO_2_) or hyperoxia (H) (85 % O_2_/5 % CO_2_) for 0, 24, 48, and 72 h. Tight junction structures (**a**, **b**, **c**, **d**; 20,000× or 80,000×) were determined by transmission electron microscopy (TEM). *Black arrows* indicate the tight junction structures (**b**, **d**). Transepithelial electrical resistance (TEER) (**e**) and apparent permeability coefficient (Papp) of fluorescein isothiocyanate–dextran (FD) (**f**) were measured. Values are represented as means ± standard deviation (SD), ^#^ *P* < 0.05 and ^##^ *P* < 0.01 for comparison between the H and N groups, ^*^ *P* < 0.05 and ^**^ *P* < 0.01 for comparison between different time points in the H group

TEM was used to visualize the TJ structures between pulmonary epithelial cells. The normoxic group had normal and intact TJ structures (Fig. 1a and b), while the hyperoxic group exhibited a loss of TJ structures (Fig. 1c) and intermittent widening (Fig. 1d) after 72 h of exposure.

Both the TEER values after hyperoxia exposure for 48 and 72 h significantly declined compared with that in the normoxic group (*P* < 0.05 for 48 h, *P* < 0.01 for 72 h). Prolonged exposure to hyperoxic conditions significantly decreased the TEER value, which reached a lowest value at 72 h (Fig. 1e).

In line with the decreased TEER values, the Papp of FD4 was higher in the hyperoxic group than in the normoxic group (*P* < 0.05 for 48 h, *P* < 0.01 for 72 h). Prolonged exposure to hyperoxic conditions significantly increased the Papp of FD4, which peaked at 72 h (Fig. 1f).

### Hyperoxia exposure decreases the expression of TJ proteins in the pulmonary epithelial barrier

**Fig. 2:**
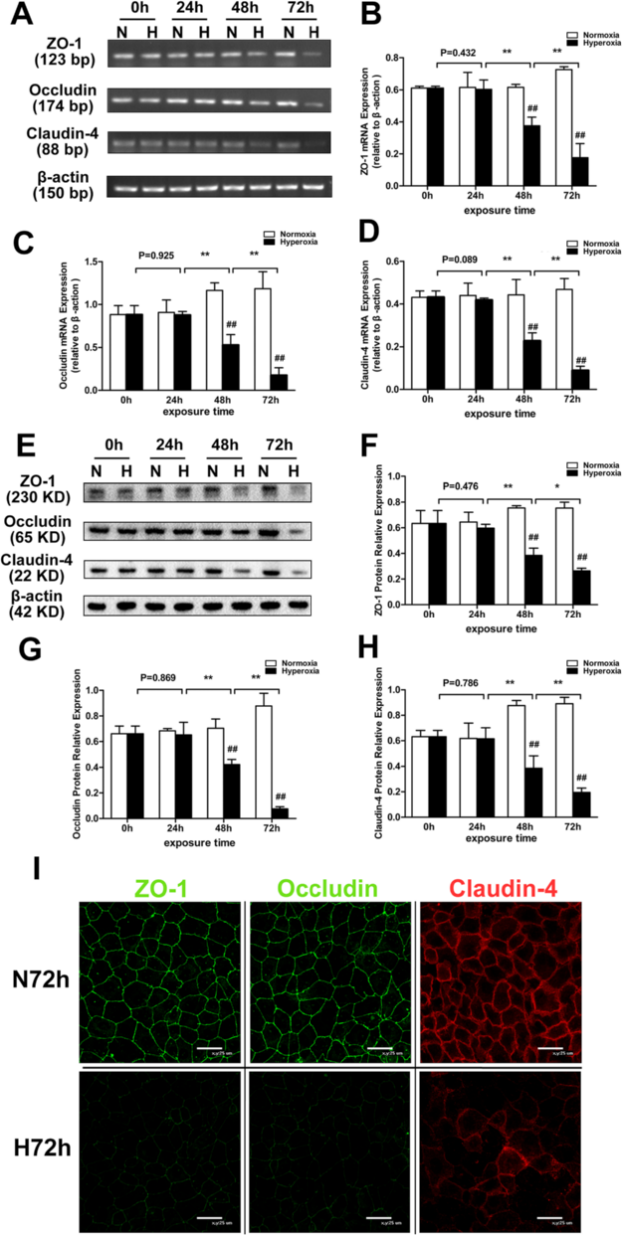
Effects of hyperoxia exposure on the expression and distribution of ZO-1, occludin, and claudin-4 in the in vitro pulmonary epithelial barrier model. Alveolar cell monolayers were exposed to normoxia (N) (21 % O_2_/5 % CO_2_) or hyperoxia (H) (85 % O_2_/5 % CO_2_) for 0, 24, 48, and 72 h. mRNA (**a**, **b**, **c**, **d**), protein (**e**, **f**, **g**, **h**), and distribution (**i**) of ZO-1, occludin, and claudin-4 were determined using RT-PCR, Western blot, and immunofluorescence staining, respectively. (**i**) *Green* indicates ZO-1 and occludin, and *red* indicates claudin-4 (800× magnification). Values are represented as means ± SD, ^##^ *P* < 0.01 for comparison between the H and N groups, ^*^ *P* < 0.05 and ^**^ *P* < 0.01 for comparison between different time points in the H group

Compared with the normoxia-treated group, both mRNA and protein levels of ZO-1, occludin, and claudin-4 decreased in the hyperoxic groups after 48 and 72 h (*P* < 0.01 for both levels). In other words, prolonged exposure to hyperoxia significantly decreased both the mRNA (Fig. 2a–d) and protein (Fig. 2e–h) levels of TJ proteins, reaching a lowest value at 72 h for both levels.

Results of the immunofluorescence staining indicated that in the normoxia-treated group, ZO-1, occludin, and claudin-4 were localized in the membranes of neighboring epithelial cells showing continuous distribution, while the fluorescence intensity decreased and distribution was discontinuous in the membrane of pulmonary epithelial cells in the 72-h hyperoxic group (Fig. 2i).

### Hyperoxia exposure decreases Cav-1 expression in the pulmonary epithelial barrier

**Fig. 3:**
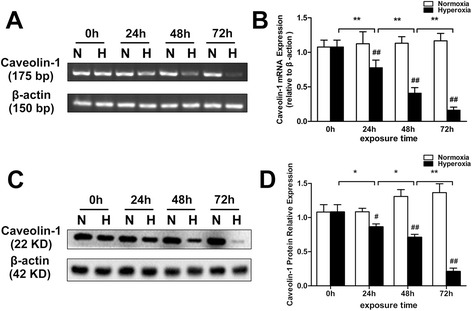
Effects of hyperoxia exposure on the mRNA and protein expression of Cav-1 in an in vitro pulmonary epithelial barrier model. Alveolar cell monolayers were exposed to normoxia (N) (21 % O_2_/5 % CO_2_) or hyperoxia (H) (85 % O_2_/5 % CO_2_) for 0, 24, 48, and 72 h. Cav-1 mRNA (**a**, **b**) and protein (**c**, **d**) levels were monitored with RT-PCR and Western blot analysis, respectively. Values are represented as means ± SD, ^#^ *P* < 0.05 and ^##^ *P* < 0.01 for comparison between the H and N groups, ^*^ *P* < 0.05 and ^**^ *P* < 0.01 for comparison between different time points in the H group

Compared with the normoxic group, *Cav-1* mRNA levels decreased in all three hyperoxia exposure groups (24, 48, and 72 h; *P* < 0.01 in all cases); similarly, the Cav-1 protein levels decreased in all three groups (*P* < 0.05 for 24 h, *P* < 0.01 for 48 and 72 h). Prolonged treatment of hyperoxia significantly decreased both the Cav-1 mRNA (Fig. 3a and b) and protein (Fig. 3c and d) levels, reaching the lowest value at 72 h for both.

### Colocalization of Cav-1 with TJ proteins in the pulmonary epithelial barrier

**Fig. 4:**
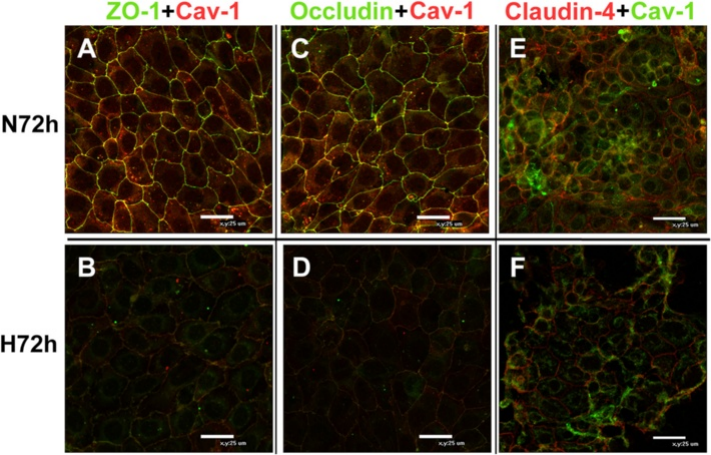
Colocalization of Cav-1 and tight junction proteins in an in vitro pulmonary epithelial barrier model. Alveolar cell monolayers were exposed to normoxia (N) (21 % O_2_/5 % CO_2_) or hyperoxia (H) (85 % O_2_/5 % CO_2_) for 72 h. The colocalization of Cav-1 and tight junction proteins in the pulmonary epithelium in vitro was monitored by double immunofluorescence staining. **a**–**d**, *green* represents ZO-1 and occludin, respectively, and *red* represents Cav-1. **e** and **f**, *green* represents Cav-1 and *red* represents claudin-4. *Yellow* represents the colocalization of Cav-1 and tight junction proteins (800× magnification)

Double immunofluorescence staining showed that Cav-1 was colocalized with TJ proteins ZO-1 (Fig. 4a and b), occludin (Fig. 4c and d), and claudin-4 (Fig. 4e and f) as shown by the yellow fluorescence in the membrane of pulmonary epithelial cells. In the normoxic group, the yellow fluorescence indicating colocalization was strong, while signal intensities of ZO-1, occludin, claudin-4, and Cav-1 were decreased after exposure to hyperoxic conditions for 72 h.

### Downregulation of Cav-1 decreases expression of TJ proteins in alveolar epithelial monolayers with normoxia exposure

**Fig. 5:**
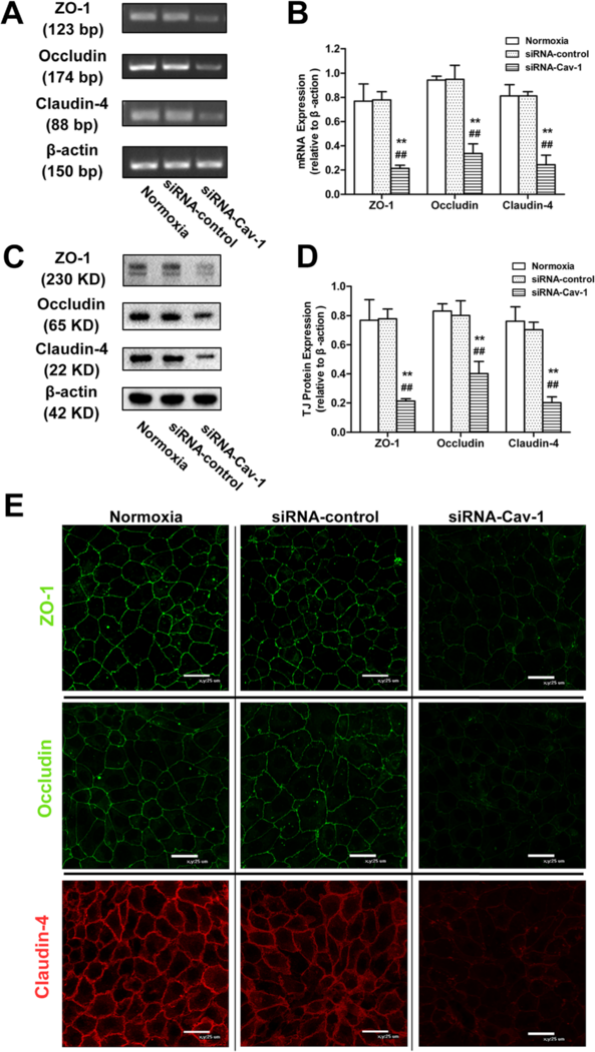
Effects of Cav-1 downregulation on the expression and distribution of ZO-1, occludin, and claudin-4 in alveolar epithelial monolayers with normoxia exposure. At 60–70 % confluence, alveolar epithelial cells were treated with normoxia exposure, control siRNA transfection, and siRNA-Cav-1 transfection (10 nm) for 72 h. mRNA (**a**, **b**), protein (**c**, **d**), and distribution (**e**) of ZO-1, occludin, and claudin-4 were monitored by RT-PCR, Western blot, and immunofluorescence staining, respectively. (**e**) *Green* represents ZO-1 and occludin, and *red* represents claudin-4 (800× magnification). Values are represented as the means ± SD, ^##^ *P* < 0.01 for comparison with the normoxia-treated group and ^**^ *P* < 0.01 for comparison with the control siRNA-transfected group

Results of the RT-PCR and Western blot analyses showed that Cav-1 mRNA and protein levels were markedly downregulated at 72 h after transfection with Cav-1-siRNA (Additional file 1: Figure S1A–H). Additional file 3 : Figure S3A-B indicated that the transfection efficiency of Cav-1-siRNA was high. Compared with the normoxic and control siRNA groups, the mRNA and protein levels of ZO-1, occludin, and claudin-4 were significantly decreased in the Cav-1-siRNA group (*P* < 0.01 for all cases; Fig. 5a–d).

Compared with the normoxic and control siRNA groups, immunofluorescence staining showed a decreased fluorescence level and discontinuous distribution of ZO-1/occludin (green) and claudin-4 (red) in the Cav-1-siRNA group (Fig. 5e).

### Downregulation of Cav-1 disrupts the structure and function of alveolar epithelial monolayers with normoxia exposure

**Fig. 6:**
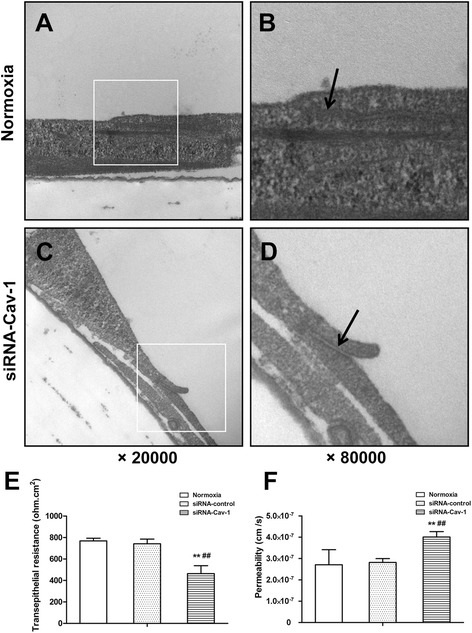
Effects of Cav-1 downregulation on the structure and function of alveolar epithelial monolayers exposed to normoxic conditions*.* At 60–70 % confluence, alveolar epithelial cells were exposed to normoxic conditions, control siRNA transfection, and siRNA-Cav-1 transfection (10 nm) for 72 h. Tight junction structures (**a**, **b**, **c**, **d**, 20,000× or 80,000× magnification) were determined by TEM. Black arrows indicate the tight junction structures (**b**, **d**). The TEER value (**e**) and Papp of FD4 (**f**) were also measured. Values are represented as means ± SD, ^##^ *P* < 0.01 for comparison with the normoxia-treated group, ^**^ *P* < 0.01 for comparison with the control siRNA-transfected group

Compared with the epithelial barrier cells of the normoxic group, which showed an intact TJ structure (Fig. 6a and b), the TJ structure was disrupted and intermittently widened between neighboring epithelial cells after Cav-1 knockdown (Fig. 6c and d).

In line with the structural disruption, Cav-1-siRNA transfection resulted in a significant reduction in the TEER value by 40 and 37 % and significant increase in the Papp of FD4 by 48 and 42 %, compared with the normoxic and the control siRNA groups, respectively (all *P* < 0.01; Fig. 6e and f).

### Upregulating Cav-1 restores expression of TJ proteins in alveolar epithelial monolayers exposed to hyperoxia

**Fig. 7:**
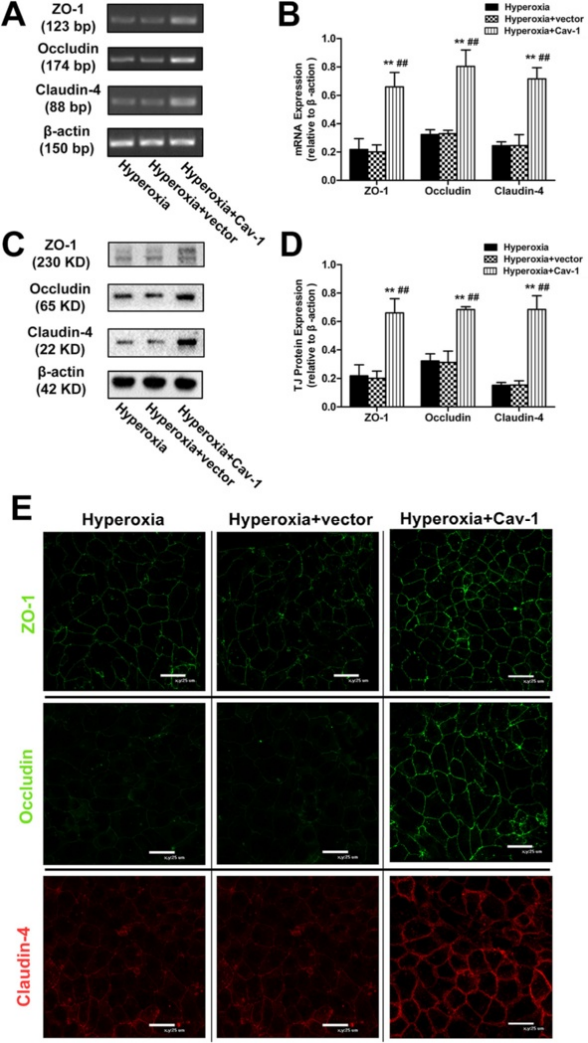
Effects of Cav-1 upregulation on the expression and distribution of ZO-1, occludin, and claudin-4 in alveolar epithelial monolayers exposed to hyperoxic conditions. At 60–70 % confluence, alveolar epithelial cells were treated with empty vector and Cav-1 cDNA (1 μg/mL) transfection and then immediately exposed to hyperoxic conditions for 48 h. mRNA (**a**, **b**), protein (**c**, **d**), and distribution (**e**) of ZO-1, occludin, and claudin-4 were monitored by RT-PCR, Western blot, and immunofluorescence staining respectively. (**e**) *Green* represents ZO-1 and occludin, and *red* represents claudin-4 (800× magnification). Values are represented as means ± SD, ^##^ *P* < 0.01 for comparison with the hyperoxia-treated group, ^**^ *P* < 0.01 for comparison with the empty vector-transfected group

Results of the RT-PCR and Western blot analysis showed that Cav-1 mRNA and protein levels were markedly upregulated at 48 h after transfection with Cav-1 cDNA (Additional file 2: Figure S2A–H). Additional file 3: Figure S3C-F indicated that the transfection efficiency of pCMV6-CAV1 under normoxic or hyperoxic condition was high. Compared with the hyperoxic and empty vector-transfected groups, upregulating Cav-1 expression increased both the mRNA and protein levels of ZO-1, occludin, and claudin-4 (*P* < 0.01 in all cases; Fig. 7a–d) under hyperoxic conditions. Accordingly, immunofluorescence staining results also showed that the distribution and fluorescence intensities of ZO-1, occludin, and claudin-4 under hyperoxic conditions were rescued (Fig. 7e) after Cav-1 cDNA transfection.

### Upregulation of Cav-1 rescues structural and functional destruction of pulmonary epithelial barrier under hyperoxic conditions

**Fig. 8:**
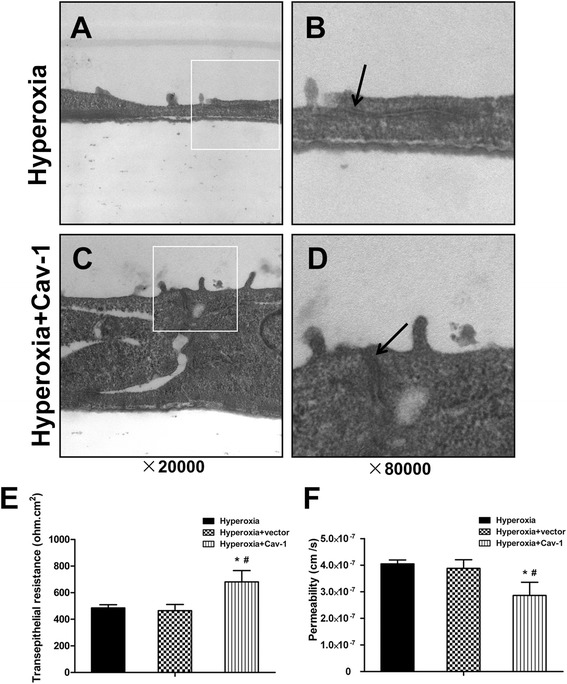
Effects of Cav-1 upregulation on the structure and function of rat alveolar epithelial monolayers exposed to hyperoxic conditions. At 60–70 % confluence, alveolar epithelial cells were treated with empty vector and Cav-1 cDNA (1 μg/mL) transfection and then immediately exposed to hyperoxic conditions for 48 h. Tight junction structures (**a**, **b**, **c**, **d**, 20,000× or 80,000× magnification) were determined by TEM. Black arrows indicate the tight junction structures (**b**, **d**). TEER value (**e**) and Papp of FD4 (**f**) were also measured. Values are presented as means ± SD, ^#^ *P* < 0.05 for comparison with the hyperoxia-treated group, ^*^ *P* < 0.05 for comparison with the empty vector-transfected group

The disruption in the TJ structure of the pulmonary epithelial barrier (Fig. 8a and b) caused by hyperoxic conditions was partially rescued and the intermittent widening was narrowed (Fig. 8c and d) after Cav-1 cDNA transfection.

In line with the structural rescue, after Cav-1 cDNA transfection, the TEER value significantly increased by 34 and 39 % and the Papp of FD4 significantly decreased by 29 and 26 %, compared with the hyperoxic and empty vector groups, respectively (*P* < 0.05 in all cases; Fig. 8e and f).

## Discussion

BPD is a common complication in preterm infants with birth weight less than 1500 g. Currently, mechanisms underlying pulmonary edema at an early stage of BPD mainly involve increased permeability of lung vasculature induced by inflammatory factors [6, 37], defects in pulmonary fluid clearance induced by aquaporin-1 and amiloride-sensitive sodium channel upregulation on the surface of AEC-II [38], and disruption of the pulmonary vascular endothelial barrier induced by downregulation of connexin proteins [8]. In our previous study, we used a hyperoxia-induced BPD model and found destruction of pulmonary epithelial TJ structures, increased permeability of the barrier, and increased wet/dry ratio of lung tissues, indicating that the destruction of the pulmonary epithelial TJ structures is a key step during the pathogenesis of pulmonary edema in BPD [7]. The results of the in vitro pulmonary epithelial barrier model exposed to hyperoxic conditions in this study are consistent with the in vivo results of our previous study. Transepithelial resistance was found to be decreased under hyperoxic exposure, which is also consistent with some in vitro experiments in which hyperoxia was found to decrease the transepithelial resistance in 16HBE14 cell and Calu-3 cell monolayers [39].

TJ is a protein complex located at the apical side of cell–cell junctions. It seals the space between neighboring cells and forms an important barrier to maintain cell polarity and modulate paracellular fluid transportation [10]. Ions, small molecules, and immune cells can transfer through the barrier and induce edema when TJ structures are broken [40, 41]. Downregulation or mislocalization of TJ proteins is known to result in pulmonary epithelial barrier destruction [13, 14]. Here, ZO-1, occludin, and claudin-4 mRNA and protein levels were found to decrease under hyperoxic conditions. Moreover, the maximum decreases in the levels of these molecules matched the maximum destruction of the pulmonary epithelial barrier structure and function, indicating that the downregulation of ZO-1, occludin, and claudin-4 is responsible for the hyperoxia-induced destruction of the pulmonary epithelial barrier. These are consistent with the in vivo results of our previous study [7].

Cav-1 is a scaffold protein with a scaffolding domain that can interact with multiple downstream signaling molecules and modulate their activities [24, 25]. Cav-1 is downregulated in several lung diseases such as asthma, chronic obstructive pulmonary disorder, and idiopathic pulmonary fibrosis [42]. In a fetal sheep lung injury model induced by in utero LPS injection, it was found that the Cav-1 gene and protein levels both decreased, and the Smad2/3, Stat, and a-SMase/ceramide signaling pathways were activated, indicating that the downregulation of Cav-1 and activation of these signaling pathways may together contribute to the occurrence of BPD [30]. In our present study, both in the hyperoxia-induced BPD model in the newborn rats (data not shown) and the in vitro pulmonary epithelial barrier model exposed to hyperoxic conditions, Cav-1 mRNA and protein levels were decreased, which is consistent with the results of the previous study [30]. Moreover, exposure to hyperoxic conditions resulted in the downregulation of Cav-1 gene transcription and protein expression levels (at 24 h after hyperoxia exposure), which preceded the downregulation of ZO-1, occludin, and claudin-4 expression at both the mRNA and protein levels (at 48 h after hyperoxia exposure). In addition, Cav-1 colocalizes with the other 3 TJ proteins in the membrane of pulmonary epithelial cells, which is consistent with some previous studies showing colocalization between Cav-1 and occludin [37]. These results reveal the existence of temporal and spatial correlations between Cav-1 and TJ proteins in the in vitro pulmonary epithelial barrier model with hyperoxia exposure.

In 2000, Nusrat and colleagues found that Cav-1 may modulate the assembly of TJ proteins [26]. In some disease models and cell lines, Cav-1 and TJ protein expression levels were either positively or negatively correlated. For instance, in a rat cortical cold injury model, the increase in Cav-1 was found to precede the decrease in the claudin-5 and occludin levels [43]. Contrarily, Song et al. found that downregulation of Cav-1 in brain microvascular endothelial cells using siRNA resulted in a concomitant decrease in the expression levels of ZO-1 and occludin [27]. Brott et al. found that ZO-1 and claudin-4 were downregulated in a rat mesenteric artery injury model induced by fenoldopam, which may have caused by the Cav-1 downregulation by the activation of nitric oxide (NO) signaling pathway [44]. In the present study, we found that Cav-1 downregulation in the pulmonary epithelial barrier model not only decreased the ZO-1, occludin, and claudin-4 mRNA and protein levels, but also disrupted the structure and function of the pulmonary epithelial barrier. Together with previous findings, our findings indicate that Cav-1 downregulation-mediated loss of TJ proteins may be the main cause of pulmonary epithelial barrier destruction induced by hyperoxia.

How Cav-1 positively regulates the expression of TJ proteins remains to be fully understood. It may be related to the activation of Src tyrosine kinases and MMPs. Src tyrosine kinases can be negatively regulated by the scaffolding domain of Cav-1 [45]. A recent study showed that the Src kinase inhibitor SU6656 can rescue the destruction of bronchial epithelial barrier and the expression of occludin [46]. Therefore, it is quite possible that Src tyrosine kinases participate in the process by which Cav-1 regulates the expression of TJ proteins. Second, some previous work showed that Cav-1 can negatively regulate MMPs [47, 48]. The activation of MMPs can lead to the degradation of TJ proteins in epithelial barriers [49, 50]. Vermeer et al. found that MMP-9 activation decreased occludin and claudin-1 in respiratory epithelium in human [20]. The downregulation of Cav-1 may lead to the activation of MMPs and further degradation of TJ proteins. However, these assumptions need further verification.

Moreover, cavtratin, a synthetic peptide with the same sequence as the scaffolding domain of Cav-1, can rescue the heightened permeability of the barrier. Cavtratin can inhibit the vascular leakage induced by carrageenan [51], decrease permeability of tumor microvasculature [52], attenuate heightened permeability of blood–brain barrier induced by monocyte chemoattractant protein-1 [27], and attenuate heightened permeability of mesenteric vessel induced by platelet-activating factor [53]. In the present study, Cav-1 upregulation was found to not only rescue the expression of TJ proteins but also to attenuate the structure and function of the in vitro pulmonary epithelial barrier exposed to hyperoxic conditions.

## Conclusions

In summary, to our knowledge, this is the first study to establish that the Cav-1-mediated downregulation of TJ proteins could be a key cause for the destruction of the pulmonary epithelial barrier when exposed to hyperoxic conditions. These results shed light into the mechanism underlying pulmonary epithelial barrier destruction during hyperoxia-induced BPD. The study suggests that Cav-1 may be a novel potential target for preventing pulmonary edema at an early stage of BPD.

## Additional files

Additional file 1: Figure S1. Effects of transfection concentration and transfection time of Cav-1 siRNA on mRNA and protein expression of Cav-1 in alveolar cell monolayers. At 72 h after transfection of Cav-1-siRNA at a final concentration of 10 nm, significant reductions in the caveolin-1 mRNA (A, B, E, F), and protein (C, D, G, H) levels were readily apparent. mRNA and protein expressions were determined by RT-PCR and Western blot analysis, respectively. β-actin was used as an internal control. Values are represented as means ± SD, ^##^ *P* < 0.01 for comparison between the normoxia and siRNA-CAV1-transfected group, ^**^ *P* < 0.01 for comparison between different concentration transfected groups or different time ransfected groups. (TIF 1844 kb) Additional file 2: Figure S2. Effects of transfection concentration and transfection time of Cav-1 cDNA on mRNA and protein expression of Cav-1 in alveolar cell monolayers. At 48 h after transfection of 1 μg/mL Cav-1 cDNA, significant increases in caveolin-1 mRNA (A, B, E, F) and protein (C, D, G, H) were readily apparent. mRNA and protein expressions were determined by RT-PCR and Western blot analysis, respectively. β-actin was used as an internal control. Values are represented as means ± SD, ^##^ *P* < 0.01 for comparison between the normoxia and CAV1 cDNA-transfected group, ^**^ *P* < 0.01 for comparison between different concentration transfected groups or different time ransfected groups. (TIF 1801 kb) Additional file 3: Figure S3. Efficacy assessment of Cav-1-siRNA transfection and pCMV6-CAV1 transfection. At 60–70 % confluence, cells exposed to normoxia were transfected with 10 nm of a control siRNA (Fluorescein Conjugate) (Cat.#6201;Cell Signaling Danvers MA) for 72 h to assess transfection efficiency for Cav-1-siRNA, while cells exposed to normoxia or hyperoxia were transfected with 1 μg/mL of pCMV6-AC-GFP (Cat.#PS100010; OriGene Technologies) for 48 h to assess transfection efficiency for pCMV6-CAV1. Images were obtained using a confocal laser scanning microscope at 100× magnification. Green represents transfected cells. The areas of transfected cells and all the cells in the view were measured by image pro plus software, and the ratio represents transfection efficiency. The transfection efficiency of control siRNA was (88.29 ± 8.25)% (A, B). The transfection efficiency of pCMV6-AC-GFP under normoxic conditions was (92.81 ± 4.16)% (C, D). The transfection efficiency of pCMV6-AC-GFP under hyperoxic conditions was (90.42 ± 6.83)% (E, F). (TIF 10078 kb)

## Abbreviations

AEC-II: type II alveolar epithelial cells
BPD: bronchopulmonary dysplasia
BSA: bovine serum albumin
Cav-1: caveolin-1
FBS: fetal bovine serum
PVDF: polyvinylidene difluoride
RT-PCR: reverse transcription-polymerase chain reaction
SD: standard deviation
SDS: sodium dodecyl sulfate
SP-C: surfactant protein C
TEER: transepithelial electrical resistance
TEM: transmission electron microscopy
TJ: tight junction
