# Supplementary figures and images for: Caveolin-1 regulates the expression of tight junction proteins during hyperoxia-induced pulmonary epithelial barrier breakdown
Source: Respir Res. 2016 May 12;17:50. doi: 10.1186/s12931-016-0364-1 (PMC4866358; doi:10.1186/s12931-016-0364-1)

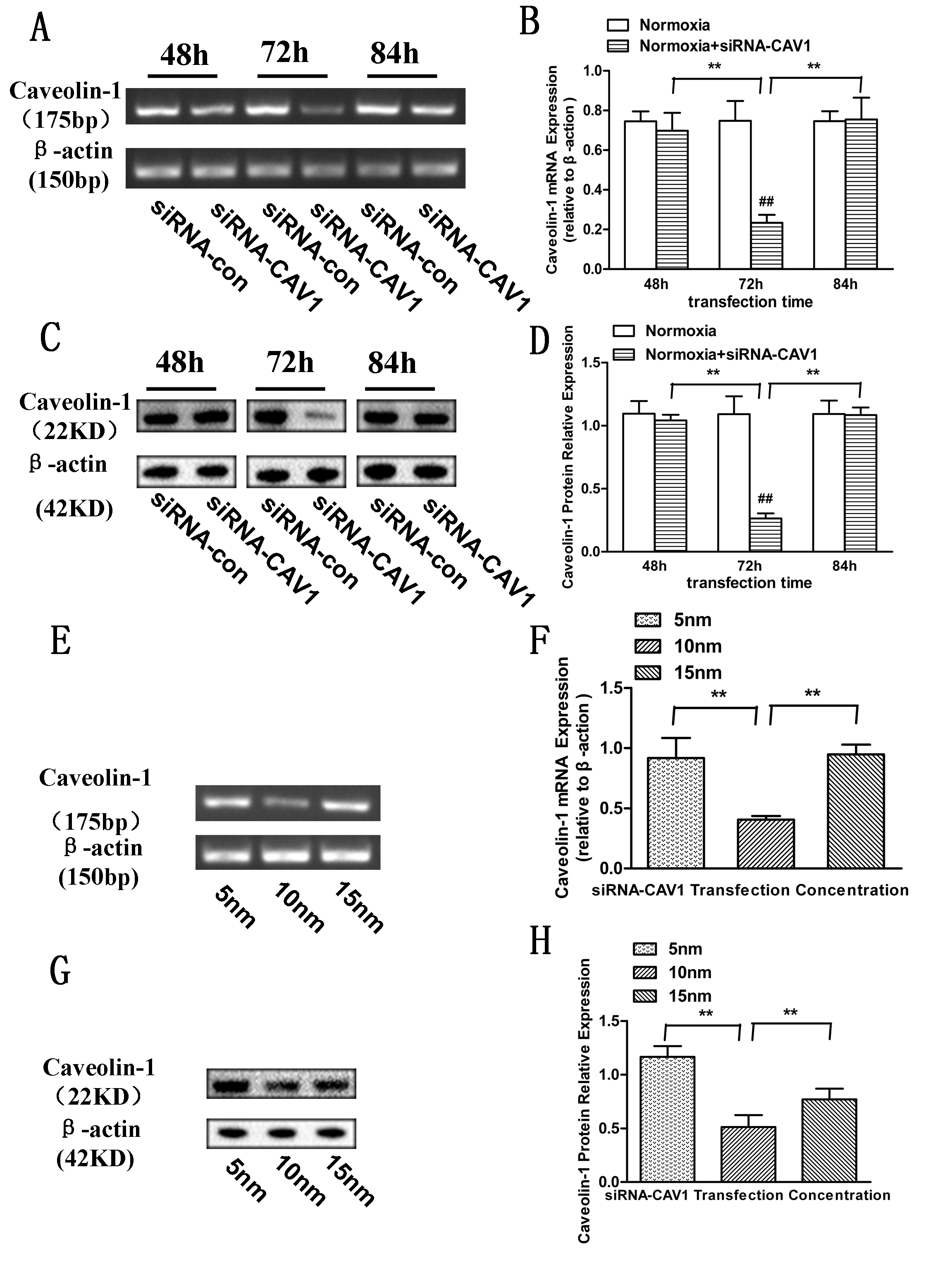

Supplement: Additional file 1: Figure S1. — Effects of transfection concentration and transfection time of Cav-1 siRNA on mRNA and protein expression of Cav-1 in alveolar cell monolayers. At 72 h after transfection of Cav-1-siRNA at a final concentration of 10 nm, significant reductions in the caveolin-1 mRNA (A, B, E, F), and protein (C, D, G, H) levels were readily apparent. mRNA and protein expressions were determined by RT-PCR and Western blot analysis, respectively. β-actin was used as an internal control. Values are represented as means ± SD, ## P < 0.01 for comparison between the normoxia and siRNA-CAV1-transfected group, ** P < 0.01 for comparison between different concentration transfected groups or different time ransfected groups. (TIF 1844 kb) [file 12931_2016_364_MOESM1_ESM.tif]

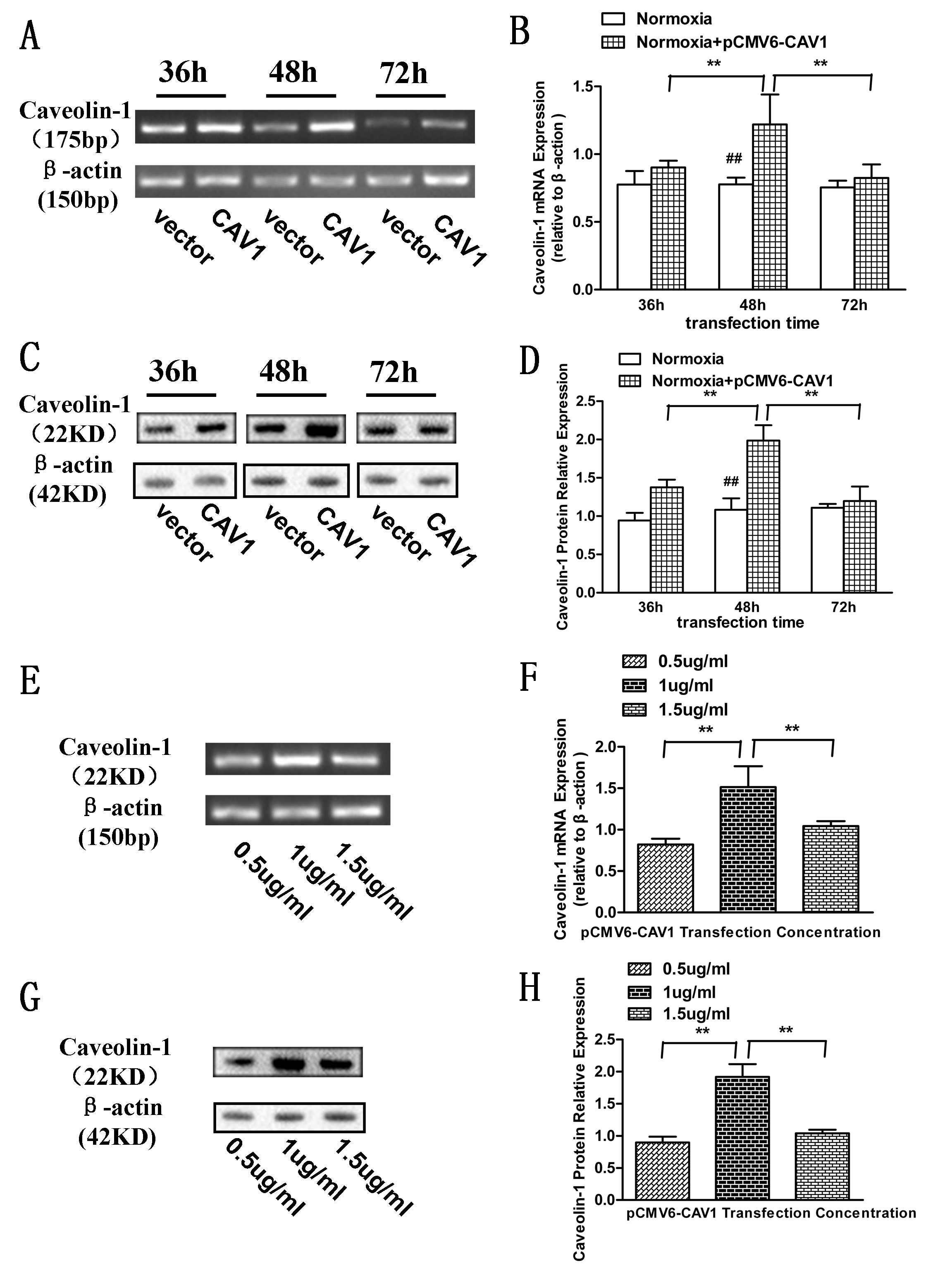

Supplement: Additional file 2: Figure S2. — Effects of transfection concentration and transfection time of Cav-1 cDNA on mRNA and protein expression of Cav-1 in alveolar cell monolayers. At 48 h after transfection of 1 μg/mL Cav-1 cDNA, significant increases in caveolin-1 mRNA (A, B, E, F) and protein (C, D, G, H) were readily apparent. mRNA and protein expressions were determined by RT-PCR and Western blot analysis, respectively. β-actin was used as an internal control. Values are represented as means ± SD, ## P < 0.01 for comparison between the normoxia and CAV1 cDNA-transfected group, ** P < 0.01 for comparison between different concentration transfected groups or different time ransfected groups. (TIF 1801 kb) [file 12931_2016_364_MOESM2_ESM.tif]

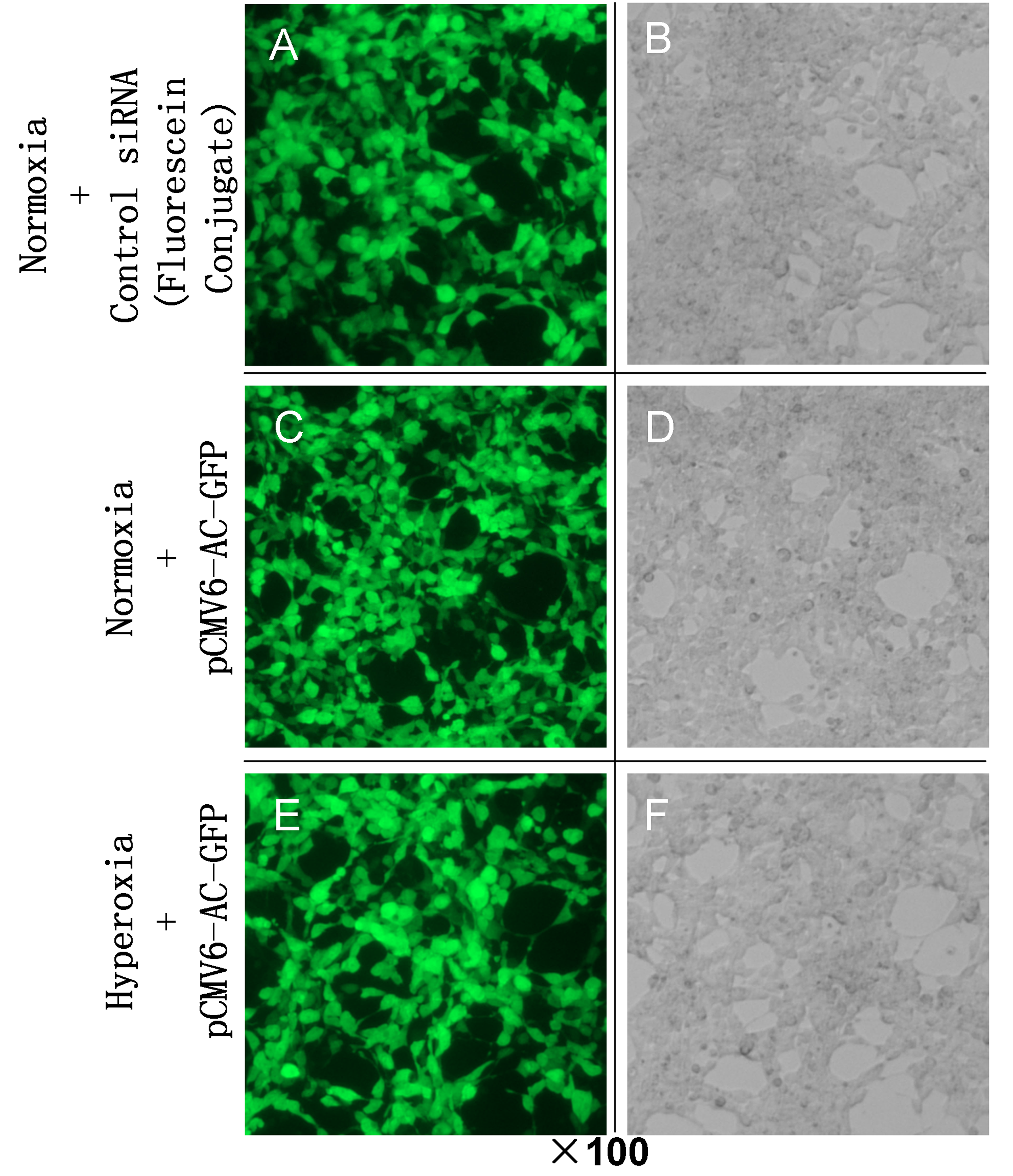

Supplement: Additional file 3: Figure S3. — Efficacy assessment of Cav-1-siRNA transfection and pCMV6-CAV1 transfection. At 60–70 % confluence, cells exposed to normoxia were transfected with 10 nm of a control siRNA (Fluorescein Conjugate) (Cat.#6201;Cell Signaling Danvers MA) for 72 h to assess transfection efficiency for Cav-1-siRNA, while cells exposed to normoxia or hyperoxia were transfected with 1 μg/mL of pCMV6-AC-GFP (Cat.#PS100010; OriGene Technologies) for 48 h to assess transfection efficiency for pCMV6-CAV1. Images were obtained using a confocal laser scanning microscope at 100× magnification. Green represents transfected cells. The areas of transfected cells and all the cells in the view were measured by image pro plus software, and the ratio represents transfection efficiency. The transfection efficiency of control siRNA was (88.29 ± 8.25)% (A, B). The transfection efficiency of pCMV6-AC-GFP under normoxic conditions was (92.81 ± 4.16)% (C, D). The transfection efficiency of pCMV6-AC-GFP under hyperoxic conditions was (90.42 ± 6.83)% (E, F). (TIF 10078 kb) [file 12931_2016_364_MOESM3_ESM.tif]
